# A prospective comparative assessment of the accuracy of the FibroScan in evaluating liver steatosis

**DOI:** 10.1371/journal.pone.0182784

**Published:** 2017-08-15

**Authors:** Baek Gyu Jun, Won Young Park, Eui Ju Park, Jae Young Jang, Soung Won Jeong, Sae Hwan Lee, Sang Gyune Kim, Sang-Woo Cha, Young Seok Kim, Young Deok Cho, Hong Soo Kim, Boo Sung Kim, So Young Jin, Suyeon Park

**Affiliations:** 1 Department of Internal Medicine, University of Ulsan College of Medicine, Gangneung Asan Hospital, Gangneung, Korea; 2 Institute for Digestive Research, Digestive Disease Center, Department of Internal Medicine, College of Medicine, Soonchunhyang University, Seoul, Korea; 3 Department of Internal Medicine, College of Medicine, Soonchunhyang University, Cheonan, Korea; 4 Department of Internal Medicine, College of Medicine, Soonchunhyang University, Bucheon, Korea; 5 Department of Pathology, College of Medicine, Soonchunhyang University, Seoul, Korea; 6 Department of Biostatistics, College of Medicine, Soonchunhyang University, Seoul, Korea; Yonsei University College of Medicine, REPUBLIC OF KOREA

## Abstract

**Background/aims:**

Recent studies have demonstrated the utility of the FibroScan^®^ device in diagnosing liver steatosis, but its usefulness has not been thoroughly appraised. We investigated the usefulness of the controlled attenuation parameter (CAP) in detecting and quantifying liver steatosis.

**Methods:**

A prospective analysis was applied to 79 chronic liver disease patients who underwent a liver biopsy, a FibroScan investigation, ultrasonography, and hepatic steatosis index (HSI). The presence and degree of steatosis as measured by the FibroScan device, ultrasonography and HSI were compared with the results for the liver biopsy tissue.

**Results:**

There was substantial concordance between the liver biopsy results and the CAP as evaluated by the kappa (κ) index test for detecting liver steatosis (κ_CAP_ = 0.77, *P*<0.001; κ_ultrasonography_ = 0.60, *P*<0.001; κ_HSI_ = 0.47, *P*<0.001). The areas under the receiver operating characteristic curve (AUROCs) of the CAP, ultrasonography, and HSI were 0.899 [95% confidence interval (CI) = 0.826–0.972)], 0.859 (95% CI = 0.779–0.939), and 0.766 (95% CI = 0.655–0.877), respectively. The optimal CAP cutoff value for differentiating between normal and hepatic steatosis was 247 dB/m, which produced sensitivity and specificity values of 91.9% and 85.7%, respectively, as well as a positive predictive value of 85.0% and a negative predictive value of 92.3%.

**Conclusion:**

The CAP produces results that are highly concordant with those of a liver biopsy in detecting steatosis. Therefore, the CAP is a noninvasive and reliable tool for evaluating liver steatosis, even in the early stages.

## Introduction

The diagnosis of hepatic steatosis is important in clinical practice for managing patients with chronic liver disease. Although hepatic steatosis is traditionally regarded as a reversible and benign condition, its role in the pathogenesis of various liver diseases has been increasingly recognized. Hepatic steatosis is associated with steatohepatitis, which can progress to liver fibrosis, cirrhosis, and even end-stage liver disease [1, 2]. In chronic hepatitis C patients, hepatic steatosis may accelerate the progression of fibrosis, have a negative effect on the rate of a sustained virological response to antiviral therapy [3–5], and be predictive of the occurrence of hepatocellular carcinoma [6]. Also, since macrovesicular steatosis of the donor liver is associated with graft failure after liver transplantation, accurately assessing hepatic steatosis is crucial in the preoperative evaluation of liver donors for transplantation [7, 8].

The current gold standard for the diagnosis and severity assessment of hepatic steatosis is a liver biopsy. However, this is invasive and prone to several complications, such as pain, bleeding, and infection, and also there is high sampling error and variability in the pathological interpretations [9–11]. Moreover, owing to the high prevalence of steatosis, its frequently benign course, and the lack of a definitive association with changes in liver enzymes, a liver biopsy can be applied only in selected patients, and moreover its invasiveness means that it cannot be repeated to monitor changes in steatosis.

These drawbacks of a liver biopsy have resulted in various noninvasive methods—especially imaging techniques such as ultrasonography, computed tomography (CT), magnetic resonance imaging (MRI), and proton magnetic resonance spectroscopy—being developed in the past decade. However, these methods also have several limitations, such as high cost in MRI and radiation exposure in CT [12, 13]. Ultrasonography is the most commonly used noninvasive imaging method for detecting hepatic steatosis [14]. Several studies have found that the sensitivity and specificity of ultrasonography in diagnosing hepatic steatosis have ranged from 60% to 94% and from 84% to 95%, respectively [12, 15–18]. However, in the morbidly obese [defined as a body mass index (BMI) of >40 kg/m^2^), its sensitivity and specificity fall to 49% and 75%, respectively [19]. While ultrasonography is simple and noninvasive, its findings are operator-dependent and it cannot be used to accurately quantify the hepatic fat content or detect a small amount of fatty infiltration [20].

To overcome these limitations, the controlled attenuation parameter (CAP) was recently introduced as a new parameter for measuring ultrasonic attenuation in the liver using ultrasonic signals acquired by the FibroScan^®^ device (Echosens, Paris, France) [21]. In a preliminary study of 115 patients with various liver disorders, Sasso et al found that the CAP showed a high accuracy for detecting steatosis, excellent performance for grading the severity of fat infiltration, and high reproducibility [22]. Many studies have demonstrated the good performance of the CAP in diagnosing and grading hepatic steatosis, but its usefulness has not been thoroughly appraised.

The aim of this prospective comparative study was to determine the usefulness of the CAP in detecting and quantifying hepatic steatosis—using a liver biopsy as the gold standard—in patients with chronic liver disease.

## Materials and methods

### Patients

This prospective observational cohort study was approved by the Institutional Review Board of Soonchunhyang University Seoul Hospital in October 2013. Of patients with chronic liver disease from various etiologies who presented at Soonchunhyang University Seoul Hospital from October 2013 to March 2017, 79 patients who met the following criteria were prospectively enrolled in the study. The inclusion criteria were as follows: (i) aged ≥19 and <70 years; (ii) providing written informed consent; (iii) the results of liver stiffness with 10 valid shots and IQR/media ratio liver stiffness <30%. The exclusion criteria were as follows: (i) malignancy of the liver or biliary tract; (ii) acute infectious disease or sepsis; (iii) chronic systemic disease, such as chronic renal failure, severe cardiovascular disease, or chronic respiratory disease; (iv) contraindications to a liver biopsy (e.g., uncontrolled bleeding tendency); or (v) factors associated with reading failure or unreliability of the FibroScan results, such as ascites, narrow rib spacing.

All 79 patients enrolled in the study underwent measurements of anthropometry (BMI; BMI = body weight (kg)/height squared (m^2^)), blood tests, liver biopsy, FibroScan investigation, and ultrasonography at an interval not exceeding 1 month. Also, hepatic steatosis index (HSI), alternative noninvasive method of assessing hepatic steatosis based on collected parameters, was calculated according to the following formulas:

HSI=8×(ALT/ASTratio)+Bodymassindex(BMI)+2(iffemale)+2(ifdiabetic)

The study was performed in accordance with the ethical guidelines of the 1975 Declaration of Helsinki.

### Liver biopsy and histological assessment

A percutaneous ultrasound-guided liver biopsy was performed using the Menghini technique by operators with at least 5 years of experience at Soonchunhyang University Seoul Hospital. Only liver biopsy specimens that were longer than 15 mm and contained at least six portal tracts were considered suitable for inclusion in the study. The obtained liver biopsy samples were fixed in formalin, embedded in paraffin, and stained with hematoxylin-eosin and Masson’s trichrome. For the purpose of the study, all of the liver biopsy specimens were analyzed by the same experienced hepatopathologist who was blinded to the clinical data of the study population.

Steatosis was graded as follows by a visual assessment based on the percentage of fat-containing hepatocytes: S0, <5%; S1, 5–33%; S2, 34–66%; and S3, >66% [23]. Liver fibrosis was categorized as follows based on the METAVIR scoring system: F0, no fibrosis; F1, portal fibrosis without septa; F2, portal fibrosis with a few septa; F3, septal fibrosis without cirrhosis; and F4, cirrhosis.

### Measurement of CAP and liver stiffness

The CAP and liver stiffness were measured using the FibroScan device by a single experienced technician who was blinded to the clinical data of the patient. The measurements were performed using a 3.5 MHz standard probe on the right hepatic lobe through the intercostal spaces with the patient lying supine. Measurements were considered valid if the following criteria were met: (i) there were at least 10 valid shots, (ii) the success rate was at least 60%, and (iii) the interquartile range was less than 30% of the median values of the CAP and liver stiffness. The final CAP and liver stiffness were recorded as the median values of all measurements, and they were expressed in dB/m and kPa, respectively [21]. The liver stiffness values were categorized as follows: F0, <5.5 kPa; F1, 5.6–7.1 kPa; F2, 7.2–9.4 kPa; F3, 9.5–12.4 kPa; and F4, ≥12.5 kPa [24].

### Ultrasonography

All of the ultrasonography examinations were performed by a single experienced investigator, and steatosis was graded as follows based on hyperechogenic liver tissue, the increased discrepancy of the echo amplitude between liver and kidney, and the loss of echoes from the walls of the portal system and diaphragm:

1. Normal—no difference in echogenicity between the liver and kidney cortex.
2. Mild—increased hepatic echogenicity with visible periportal and diaphragmatic echogenicity.
3. Moderate—increased hepatic echogenicity with imperceptible periportal echogenicity without obscuration of the diaphragm.
4. Severe—increased hepatic echogenicity with imperceptible periportal echogenicity and obscuration of the diaphragm [25].

### Statistical analyses

Data are expressed as number (percentage) or mean ± standard deviation values. The sensitivity, specificity, positive predictive value (PPV), and negative predictive value (NPV) of the CAP, ultrasonography, and HSI were calculated. The accuracy of steatosis diagnoses was determined by computing Cohen’s kappa (κ) index test values and the areas under the receiver operating characteristic curve (AUROCs). The optimal cutoff value that maximized the accuracy of the CAP for diagnosing significant steatosis was calculated using the Youden index. κ agreement values were interpreted in the following way: poor (0.00–0.20), fair (0.21–0.40), moderate (0.41–0.60), substantial (0.61–0.80), and good (0.81–1.00) [26]. Box plots were used to show the CAP distributions according to histological steatosis grade. Univariate and multivariate logistic regression analyses were conducted for evaluating factors associated with steatosis on liver biopsy. Statistical analyses were performed using SPSS software (version 20.0, SPSS, Armonk, NY, USA) and R software (version 3.3.1) with the pROC and Optimal Cut Points packages (R Development Core Team 2015). A *P* value less than 0.05 was considered statistically significant.

## Results

### Patient characteristics

The baseline characteristics of the 79 patients are summarized in Table 1. Their mean age was 46.1 years, and 30 of the patients were male. The etiologies of the chronic liver diseases were alcoholic liver disease (*n* = 9), chronic hepatitis B (*n* = 12), chronic hepatitis C (*n* = 10), nonalcoholic fatty liver disease (NAFLD) (*n* = 42), and unknown hepatitis (*n* = 6). The mean BMI was 26.0 kg/m^2^.

**Table 1:** Clinical characteristics of the patients.

Characteristics				Patient data (n = 79)
Age (years)				46.1±14.8
Gender				
	Male			30
	Female			49
Hypertension				15
Diabetes mellitus				20
Chronic liver disease etiology				
		Alcoholic liver disease		9
		Chronic hepatitis B		12
		Chronic hepatitis C		10
		NAFLD		42
		others		6
Body mass index (kg/m^2^)				26.0±5.1
Waist circumference (cm)				87.7±21.3
Total cholesterol (mg/mL)				175.2±38.5
Triglyceride (mg/mL)				148.6±112.7
HDL-cholesterol (mg/mL)				48.7±16.8
LDL-cholesterol (mg/mL)				105.1±38.8
AST (IU/L)				68.0±51.0
ALT (IU/L)				76.0±71.8
Total bilirubin (mg/dL)				0.8±1.1
Serum albumin (g/dL)				4.1±0.4
Platelet (10^3^/μL)				187.5±65.7
PT(INR)				1.0±0.1
Liver histology				
		Steatosis	S0/S1/S2/S3	41/22/12/4
		Metavir score	F0/F1/F2/F3/F4	10/22/23/15/9
Ultrasonography				
	normal/mild/moderate/severe			32/21/15/11
CAP (dB/m, mean±SD)				251.1±65.8
Liver stiffness value (kPa, mean±SD)				12.8±10.6

NAFLD, non alcoholic fatty liver disease; AST, aspartate aminotransferase; ALT, alanine aminotrasferase; PT, prothrombin time; HDL, high-density lipoprotein; LDL, low-density lipoprotein; CAP, controlled attenuation parameter; SD, standard deviation

The distributions of the steatosis grade according to the results of liver biopsy and ultrasonography are presented in Table 1. The numbers of patients with histological grades of S0, S1, S2, and S3 were 41 (51.9%), 22 (27.8%), 12 (15.2%), and 4 (5.1%), respectively. The ultrasonography examinations revealed that 32 (40.5%) patients were normal, while mild, moderate, and severe steatosis was present in 21 (26.6%), 15 (19.0%), and 11 (13.9%) patients, respectively. The mean CAP value was 251.1 dB/m.

### Diagnostic performances of CAP, ultrasonography and HSI

The diagnostic performances of the CAP, ultrasonography and HSI for steatosis are outlined in Table 2. The sensitivity, specificity, PPV, and NPV of the CAP were 91.9%, 85.7%, 85.0%, and 92.3%, respectively. The estimated κ value for the association between liver biopsy and the CAP was 0.772 (*P*<0.001), which was higher than those for ultrasonography (0.600, *P*<0.001) and HSI (0.470, *P<*0.001), which showed the overall good concordance between liver biopsy and the CAP for diagnosing steatosis.

**Table 2:** Diagnostic performance of the CAP and ultrasonography.

						McNemar	Kappa index test		
	sensitivity	specificity	PPV	NPV	AUROC	*P-value*	estimate	*P-value*	agreement
CAP	0.919	0.857	0.850	0.923	0.899	0.505	0.772	<0.001	substantial
USG	0.919	0.690	0.723	0.906	0.859	0.024	0.600	<0.001	moderate
HSI	0.784	0.690	0.690	0.784	0.766	0.383	0.470	<0.001	moderate

CAP, controlled attenuation parameter; USG, ultrasonography; HSI, hepatic steatosis index; PPV, positive predictive value; NPV, negative predictive value; AUROC, area under the receiver-operator curve

AUROCs are given with 95% confidence interval (95% CI)

### Comparison of CAP, ultrasonography and HSI for detecting hepatic steatosis

**Fig 1:**
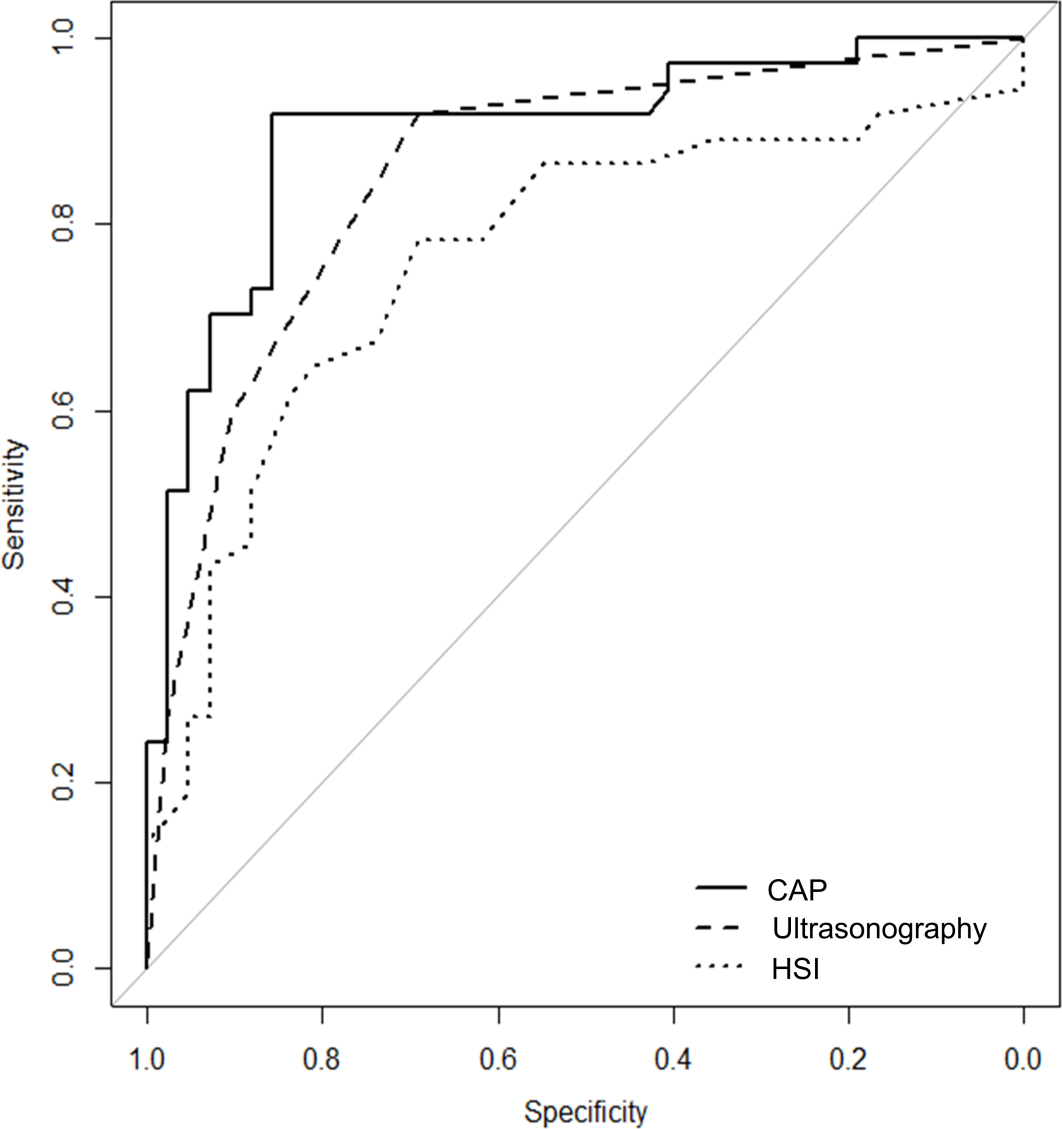
ROC curves and AUROC for CAP, ultrasonography and HSI for detecting hepatic steatosis. ROC, Receiver operating characteristics (ROC); AUROC, and area under ROC; CAP, Controlled attenuation parameters, HSI, hepatic steatosis grade.

The CAP is useful for detecting steatosis. The high diagnostic accuracy of the CAP for steatosis was also shown by the ROC curve and the AUROC (Table 2 and Fig 1). The AUROC of the CAP was 0.899 [95% confidence interval (CI) = 0.826–0.972], which is higher than the value for ultrasonography and HSI, at 0.859 (95% CI = 0.779–0.939) and 0.766 (95% CI = 0.655–0.877), respectively. The CAP has higher diagnostic accuracy than HSI (*P* = 0.039)

### Relationship between CAP and steatosis grade

**Fig 2:**
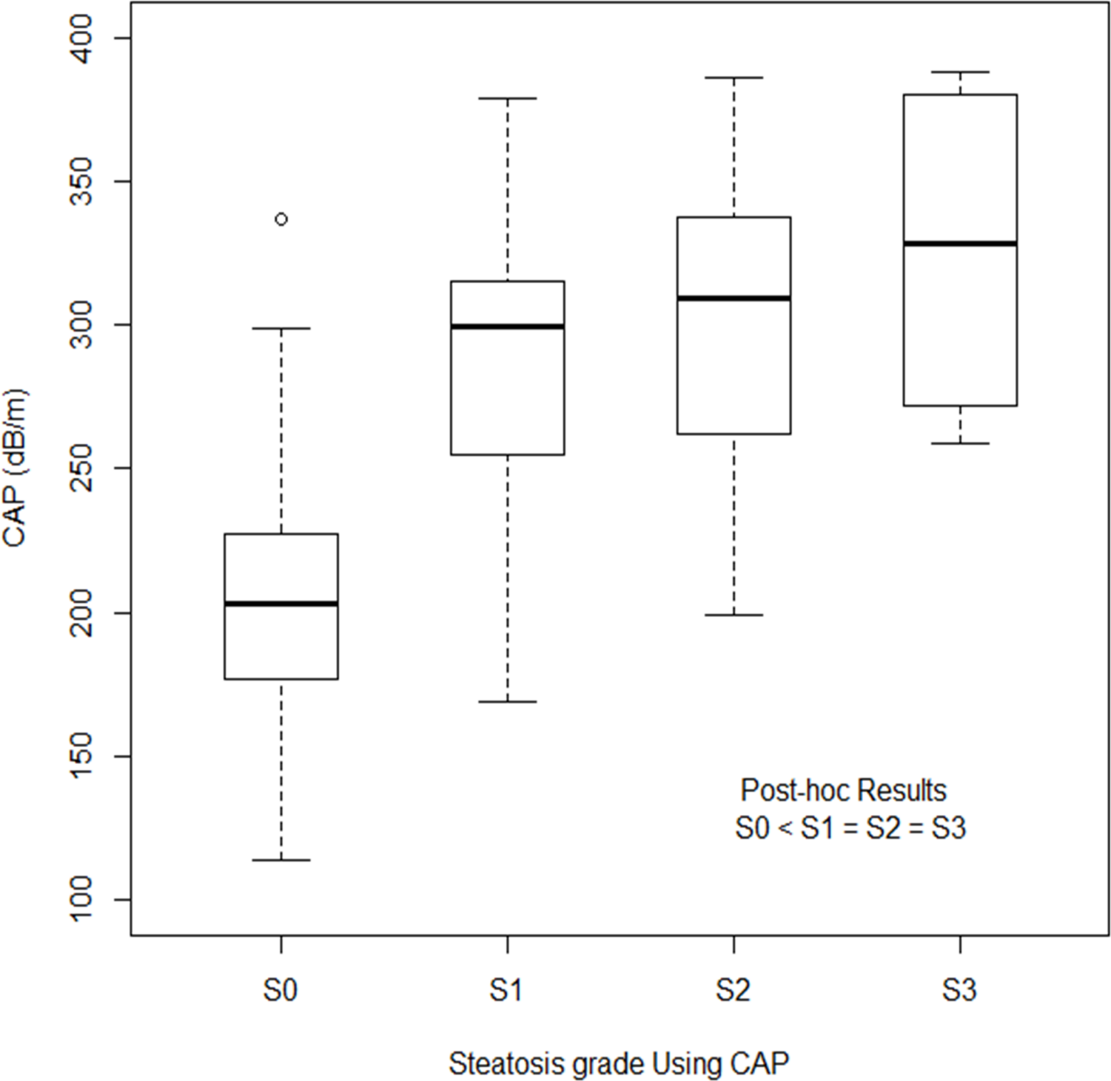
The distribution CAP values according to histologic steatosis grade. The line through the box indicates the median. The bottom and top of each box represent the 25th and 75th percentiles. CAP was only significantly different between S0 and S1 (P<0.001), while difference between S1 and S2 (P = 1.000), and S2 and S3 (P = 1.000) was not significant. CAP, Controlled attenuation parameters.

The CAP is useful in quantifying steatosis. The estimated κ value for the association between liver biopsy and the CAP was 0.772 (*P<*0.001), which represented substantial agreement. The CAP exhibited good performance in detecting early-stage steatosis. The CAP differed significantly only between patients at grades S0 and S1 (*P*<0.001), and not between the other steatosis grades (Fig 2). The median CAP values for patients with steatosis of grades S0, S1, S2, and S3 were 203.0 dB/m (range = 114.0–337.0dB/m), 299.5 dB/m (range = 169.0–379.0 dB/m), 309.5 dB/m (range = 199.0–386.0 dB/m), and 329.5 dB/m (range = 259.0–388.0 dB/m), respectively.

### Optimal cutoff value of the CAP

The optimal CAP cutoff value for differentiating between normal and hepatic steatosis (≥S1) was 247 dB/m (AUROC = 0.899, 95% CI = 0.826–0.972), which produced a sensitivity and specificity of 91.9% and 85.7%, respectively, as well as a PPV of 85.0% and a NPV of 92.3%. Meanwhile, to facilitate clear interpretation, we considered CAP values of <200 dB/m and >300 dB/m, which showed 94.6% sensitivity in CAP <200 dB/m and 100.0% specificity in CAP>300 dB/m, respectively (Table 3). This suggests that the CAP can be reliably used to determine whether or not hepatic steatosis is present.

**Table 3:** The optimal cut off value of the CAP for differentiating between normal and hepatic steatosis.

						McNemar	Kappa index test		
	sensitivity	specificity	PPV	NPV	AUROC	*P-value*	estimate	*P-value*	agreement
CAP (200)	0.946	0.405	0.583	0.895	0.675	0.000	0.338	0.001	fair
CAP (300)	0.514	1.000	0.950	0.695	0.745	0.000	0.503	0.001	moderate

CAP, controlled attenuation parameter; PPV, positive predictive value; NPV, negative predictive value; AUROC, area under the receiver-operator curve

### Factors associated with steatosis on liver biopsy

Clinical and biological factors associated with steatosis were evaluated. As shown in Table 4, univariate analysis identified that age >50 year, BMI and NAFLD were significant factors. By multivariate analysis, factors associated with steatosis were BMI > 30 kg/m^2^ and NAFLD.

**Table 4:** Factors associated with steatosis on liver biopsy.

	Univariate analysis			Multivariate analysis		
	Odds ratio	95% CI	P value	Odds ratio	95% CI	P value
Sex (Male)	0.713	0.286–1.774	0.467			
Age >50 year	0.399	0.159–0.999	0.050	0.327	0.086–1.246	0.102
Diabetes	0.221	0.679–5.342	1.904			
Hypertension	0.552	0.176–1.735	0.309			
Chronic hepatitis B	0.311	0.163–1.782	0.311			
Chronic hepatitis C	0.999	0.000–0.000	1.000			
NAFLD	10.826	3.476–30.436	<0.001	8.920	2.303–34.554	0.002
Alcohol	1.919	0.426–8.648	0.396			
ALT >80IU/ml	2.145	0.771–5.964	0.144			
BMI						
[25–30] vs.≤25 kg/m^2^	4.284	1.396–13.148	0.011	3.739	0.981–14.250	0.053
>30 vs. [25–30] kg/m^2^	61.625	7.083–536.150	<0.001	8.920	3.636–341.834	0.002

NAFLD, non alcoholic fatty liver disease; CI, confidence interval; ALT, alanine aminotrasferase; BMI, body mass index.

### Diagnostic performance of liver stiffness measurements

The κ index test indicated that the diagnostic performance of hepatic fibrosis measurements is moderate. The sensitivity, specificity, PPV, and NPV of measurements using the FibroScan device were 92.0%, 63.0%, 53.5%, and 94.4%, respectively.

## Discussion

The CAP is now widely used as a noninvasive, objective, and safe method for assessing steatosis [22], and it has been shown to be effectively at detecting early-stage steatosis (>5% steatosis), and it is also feasible in irrespective of age, even for children [27, 28]. Although the CAP is used for detecting steatosis and measuring the steatosis grade, there have been few attempts to validate its use. Previous studies have found that the CAP is better at detecting steatosis than determining the steatosis grade.

The present study found that the performance of the CAP in detecting steatosis was acceptable, with good accuracy (AUROC = 0.899 for ≥S1) (Table 2), which is consistent with previous studies. Lee et al [29] proposed that the CAP exhibited good accuracy, with AUROC values of 0.953 for ≥S1, 0.855 for ≥S2, and 0.726 for S3; Chon et al [30] reported similar results, with corresponding values of 0.885, 0.894, and 0.800. Another study found that AUROC and the accuracy of the CAP in chronic hepatitis C patients are satisfactory for detecting >10% steatosis [31]. Based on our data we concluded that the CAP has good accuracy in detecting early-stage hepatic steatosis (>5%).

In this study, factors associated with steatosis on liver biopsy were investigated. BMI>30 kg/m^2^and NAFLD were independently associated with steatosis. The influence of BMI has already been reported in several studies [30, 32]. An interesting result is that only NAFLD is associated with steatosis in etiologic factors, while viral hepatitis and alcohol abuse were not. Further studies are necessary to validate the influences related to etiologies.

The quantification of hepatic steatosis is still a matter of debate. Several studies have found the CAP to be correlated with the histological steatosis grade, but that differentiating the steatosis grade was not satisfactory. In our study, the CAP showed poor accuracy for differentiating the steatosis grade—differentiation was only possible between grades S0 and S1, while there was no significant difference between grades S1 and S2 or between grades S2 and S3 (Fig 2). Similar studies have shown limitations in differentiating late-stage steatosis or steatosis of adjacent grades. In particular, most studies have found it difficult to separate grades S2 and S3 [22, 30, 31, 33–35]. Chon et al reported that separating the steatosis grade is possible in early-stage steatosis [30]. Sasso et al suggested that the CAP is good for differentiating between large differences in steatosis grades [31]. Myers et al [33] and Sasso et al [22] also reported that the CAP could not separate steatosis grades S2 and S3, although the CAP was correlated with the steatosis grade. Moreover, high CAP values are associated with a high risk of discordance between liver biopsy and CAP findings [36]. Fujimori et al have shown limitation that the meaningful positive correlation was not found in the patients with BMI of 25 kg/m^2^ or more or stage 2–4 fibrosis [37]. We therefore suggest that while the CAP is positively correlated with the steatosis grade, it cannot be used to accurately differentiate steatosis grades in the late-stage steatosis or obese patients. To overcome these limitations, further studies are needed for accurate steatosis quantification.

The cutoff value of CAP for the diagnosis of hepatic steatosis is still controversial, with it varying with the study population and steatosis criteria. In the present study we found that the optimal cutoff value of CAP for detecting hepatic steatosis of >5% was 247 dB/m (AUROC = 0.899, 95% CI = 0.826–0.972). The sensitivity and specificity values of the CAP were 91.9% and 85.7%, respectively. This finding is similar to previous studies finding optimal cutoff values for grade S1 (5–33% steatosis) of 247 dB/m [29] and 250 dB/m [30]. However, the determined cutoff value has differed markedly between studies. Lédinghen et al [38] and Sasso [31] reported low cutoff values for detecting ≥S1 steatosis (which is defined as a percentage of hepatocytes with a fat content of >11%) were 214 and 222 dB/m, respectively. In contrast with these studies, Myers et al [33] suggested a higher cutoff value of 283 dB/m. Previous studies on the accuracy and cutoff values of CAP in discriminating steatosis are summarized in Table 5.

**Table 5:** Summary of previous studies on the accuracy and cutoff values of CAP in discriminating steatosis.

Author, year (Reference)	Patients (n)	Etiology (n)	Steatosis definition	Cut off of steatosis	AUROC	Sensitivity	Specificity
Sasso et al (2010) [22]	115	-	Steatosis ≥ 11%	237.7 dB/m	0.91	91%	81%
Meyers et al (2012) [33]	153	Viral hepatitis (67)	Steatosis ≥ 10%	283 dB/m	0.81	76%	79%
		NAFLD (72)					
		Others (9)					
Le´dinghen et al (2012) **[38]**	112	NAFLD (28)	Steatosis ≥ 11%	215 dB/m	0.84	≥ 90%	-
		HCV (40)					
		Alcohol (6)					
		Others (38)					
Sasso et al (2012) [31]	615	HCV (615)	Steatosis ≥ 11%	222 dB/m	0.80	76%	71%
Chon et al (2014) [30]	135	NAFLD (56)	Steatosis ≥ 5%	250 dB/m	0.88	73%	95%
		HBV (47)					
		HCV (12)					
Lee et al (2016) [29]	183	NAFLD (94)	Steatosis ≥ 5%	247 dB/m	0.85	88%	100%
		Non-NAFLD (89)					

NAFLD, non alcoholic fatty liver disease; HBV, hepatitis B virus; HCV, hepatitis C virus

We analyzed the sensitivity and specificity when using CAP cutoff values of 200 and 300 dB/m, respectively (Table 3). When the cutoff was 200 dB/m, the sensitivity was 94.6% and AUROC was 0.675; that is, there was few possibility of steatosis when the CAP was ≤200 dB/m. Meanwhile, when the cutoff was 300 dB/m, the specificity was 100% and AUROC was 0.745; that is, all cases had steatosis when the CAP was ≥300 dB/m. The correlation between CAP value and the severity of steatosis was not clear for values between 200 and 300 dB/m, and so further study is needed to clarify this. Finally, our results showed that a cutoff of 247 dB/m was the most appropriate for diagnosing hepatic steatosis.

We compared the usefulness of the CAP, ultrasonography, and HSI for detecting hepatic steatosis. The AUROC value for the diagnostic performance for steatosis was higher for the CAP (0.899) than for ultrasonography (0.859) and HSI (0.766) (Table 2). The CAP only showed substantial agreement in diagnostic performance in detecting steatosis. However, ultrasonography and HSI were moderate agreement. These results suggest that the CAP is the most accurate among the three examinations for detecting hepatic steatosis.

In comparison with other modalities, the CAP presents several advantages compared to ultrasonography and HSI. First, the CAP can be used to detect early-stage steatosis; in this study the CAP demonstrated good accuracy for detecting >5% steatosis. Lédinghen et al suggested that the CAP is very effective for detecting even low-grade steatosis [38]. In contrast, it is difficult to detect a fatty liver early using ultrasonography. Previous meta-analyses that have assessed the performance of ultrasonography found that its sensitivity increases with increasing degree of fatty infiltration [39]. Ultrasonography allows for the reliable detection of a moderate-to-severe fatty liver (≥20% steatosis) with a sensitivity of 84.8% and a specificity of 93.6% [14]. Second, the CAP is an objective and reproducible method with a low interobserver variability [33, 40]. In contrast, the reproducibility of diagnoses of fatty liver using ultrasonography is poor because the procedure is highly dependent on both the operator and the device used [41]. HSI also showed objective results, but low diagnostic accuracy adjusted by Bonferroni correction compared to CAP (*P* = 0.020). Third, the CAP can be performed using simultaneous measurements of liver stiffness and steatosis. Moreover, the diagnostic accuracy and quantification of hepatic fibrosis has been widely validated [42]; we also showed a moderate diagnostic performance for the liver stiffness in this study.

Our study was subject to several limitations. First, although this study had a prospective design, the overall sample was small, especially for steatosis of grades S2 and S3. This made it difficult to identify differences between late-stage steatosis. Small sample might be related to the false negative results regarding the accuracy of CAP in discriminating late stage steatosis. Second, ultrasonography is performed by a single experienced investigator in this study. Considering that ultrasonographic findings are basically operator-dependent, at least two investigators would have been necessary for more reliable grading of steatosis. To minimize the possibility of intra-observer bias, we performed ultrasonographic examination twice during hospitalization, before and after liver biopsy.

## Conclusions

This study has demonstrated that the CAP has a high concordance with the results of a liver biopsy in detecting steatosis. The CAP is moderately correlated with the steatosis stage, although it cannot be used to differentiate adjacent steatosis stages. The CAP is a noninvasive and reliable tool for evaluating liver steatosis, even in the early stages.

## Supporting information

S1 Data File This file provides the data of manuscript. (XLSX)
